# Standard and increased canakinumab dosing to quiet macrophage activation syndrome in children with systemic juvenile idiopathic arthritis

**DOI:** 10.3389/fped.2022.894846

**Published:** 2022-07-28

**Authors:** Mikhail M. Kostik, Eugenia A. Isupova, Konstantin Belozerov, Tatyana S. Likhacheva, Evgeny N. Suspitsin, Rinat Raupov, Vera V. Masalova, Irina A. Chikova, Margarita F. Dubko, Olga V. Kalashnikova, Vyacheslav G. Chasnyk, Randy Q. Cron

**Affiliations:** ^1^Hospital Pediatry, Saint-Petersburg State Pediatric Medical University, Saint-Petersburg, Russia; ^2^Medical Genetics, Saint-Petersburg State Pediatric Medical University, Saint-Petersburg, Russia; ^3^Molecular Oncology, National Medical Research Center of Oncology n.a. N.N. Petrov, Saint-Petersburg, Russia; ^4^Division of Rheumatology, Department of Pediatrics, University of Alabama School of Medicine, Birmingham, AL, United States

**Keywords:** canakinumab, interleukin-1, macrophage activation syndrome, monoclonal antibody, dosing, systemic juvenile idiopathic arthritis

## Abstract

**Objective:**

Macrophage activation syndrome (MAS) is a life-threatening, potentially fatal condition associated with systemic juvenile idiopathic arthritis (sJIA). Interleukin-1 (IL-1) is a key cytokine in the pathogenesis of sJIA MAS. Many cases of MAS are medically refractory to traditional doses of biologic cytokine inhibitors and may require increased dosing. When MAS occurs in the setting of sJIA treated with the IL-1 receptor antagonist (IL-1Ra), anakinra, increased anakinra dosing may be beneficial. Increased dosing of another IL-1 inhibitor, canakinumab, a monoclonal antibody to IL-1β, has not been reported to treat refractory MAS in the setting of sJIA.

**Methods:**

Retrospective data collection extracted from the electronic medical record focused on canakinumab usage and dosing in 8 children with sJIA who developed MAS at a single academic center from 2011 to 2020.

**Results:**

Eight sJIA children (five girls) with median age 8.5 years (range, 0.9–14.2 years) were included in the present study. Five children developed MAS at disease onset and three during ongoing canakinumab therapy. MAS resolved in all eight children with canakinumab treatment. When the canakinumab dosing was insufficient or MAS developed during canakinumab therapy, the dosing was temporally up-titrated (four patients, maximum 300 mg per dose) without observed side effects.

**Conclusion:**

This report provides evidence for the efficacy and safety of short-term increased doses (2–3-times normal) of canakinumab in treating sJIA associated MAS. Further study of the efficacy and safety of increased doses of canakinumab for treatment of MAS in children with sJIA is warranted.

## Introduction

Macrophage activation syndrome (MAS) is a sometimes fatal complication of rheumatic disorders, most commonly seen in systemic juvenile idiopathic arthritis (sJIA) in childhood (1, 2). MAS belongs to the H group of histiocytic disorders, which also includes familial hemophagocytic lymphohistiocytosis (HLH), which has been traditionally treated with high doses of etoposide chemotherapy (3). Since MAS is not considered to be a result of homozygous mutations in cytolytic pathway genes (4), and because the HLH treatment protocol is associated with a high degree of mortality, rheumatologists have chosen to treat rheumatic disease associated MAS with non-cytotoxic immunosuppression. This typically includes a combination of high dose corticosteroids (CS), the calcineurin inhibitor, cyclosporine A, and, more recently, the addition of the recombinant interleukin-1 (IL-1) receptor antagonist (IL-1Ra), anakinra (2). While lower doses of anakinra (1–2 mg/kg/day) may suffice to treat MAS associated with sJIA (5), when patients are already taking anakinra, even higher doses may be required to control MAS (6). In addition to anakinra, other IL-1 blocking agents are available for treatment of sJIA (7). Canakinumab is a monoclonal antibody directed against IL-1β and has been shown to be highly effective in treating sJIA (8). Data about the efficacy of canakinumab in MAS associated with sJIA is scarce, and when MAS occurs in the setting of canakinumab, it is currently unknown whether increased dosing of canakinumab will help treat the MAS. Thus, the objective was to evaluate and report on the potential benefit of increased canakinumab dosing to resolve MAS in the setting of sJIA.

## Materials and methods

### Patients and data collection

Treatments and laboratory results, as well as demographic data and clinical features of nine children diagnosed with sJIA and MAS treated with canakinumab at Saint-Petersburg State Pediatric Medical University Children's Hospital between 2011 and 2020, were collected and reviewed retrospectively. The diagnosis of sJIA was based on the clinical and laboratory features according to the 2004 revised ILAR classification criteria (9). MAS was diagnosed according to the 2016 international consensus classification criteria (10). The number of systemic features was calculated according to published consensus opinion (11). The clinical and laboratory features of sJIA and MAS during disease onset and MAS episodes, as well as during canakinumab treatment, were recorded. Mean values and confidence intervals were calculated using GraphPad Prism software (San Diego, CA, USA). Clinical remission was determined according to the Wallace JIA remission criteria (12). Adverse events were identified as previously described (13). The study was approved by the Ethics Committee of Saint-Petersburg State Pediatric Medical University. Specifically, approval of higher-dose canakinumab treatment was obtained from the local hospital authorities at Saint-Petersburg State Pediatric Medical University according to Russian Federal Medical Law. Deviation from medical permission to use maximum doses (300 mg) of canakinumab did not occur.

## Results

From 2011 through 2020, 8 sJIA patients (five girls) with median age 8.5 years (range, 0.9–14.2 years) developed MAS and were treated with canakinumab, out of 104 sJIA patients in the local cohort. The main clinical features at disease onset included the following: fever - 8 (100%), active arthritis - 4 (50%), pleuritis - 5 (62.5%), pericarditis - 4 (50%), peritonitis - 2 (25%), rash - 7 (87.5%), hepatomegaly - 8 (100%), splenomegaly - 8 (100%), and lymphadenopathy - 6 (75%). The remaining five patients without active arthritis at onset had initial muscle and joint pain and developed arthritis later; the longest period was 22 months. Five patients developed MAS during disease onset and three developed MAS during canakinumab treatment. Patients with MAS had intractable fever – 8 (100%), hepatosplenomegaly – 8 (100%), serositis – 5 (62.5), bleeding - 3 (37.5%), and CNS involvement - 2 (25%). Initial treatment included high doses pulse therapy with methylprednisolone - 7 (87.5%), followed by oral corticosteroids (prednisolone or methylprednisolone, 1–2 mg/kg/day) - 8 (100%). In some patients, additional pulses of high-dose (30 mg/kg, maximum one gram daily) methylprednisolone were given for persistent fever and progressive features of MAS. Methotrexate was given to four patients (50%), cyclosporine A to three patients (37.5%), and intravenous immunoglobulin (IVIG) to five patients (62.57%). The initial biologic therapy was tocilizumab (anti-IL-6 receptor monoclonal antibody) in three patients (37.5%) and canakinumab in five (62.5%). The demographic features, laboratory values, and biologic treatments of the patients are listed in Table 1.

**Table 1:** Demographic, clinical, and laboratory features of sJIA patients during the MAS episodes.

**Parameter**	**Results** ***n*** **(%) or mean and CI (25%; 75%)**
Females/males, *n* (%)	5 (62.5)/3 (37.5)
Onset age, years	8.5 (6.6; 11.2)
MAS at onset, *n* (%)	5 (62.5)
Number of systemic features, *n* (%): Fever Pleuritis Pericarditis Peritonitis Rash Hepatomegaly Splenomegaly Lymphadenopathy	8 (8, 9) 8 (100%) 5 (62.5%) 4 (50%) 2 (25%) 7 (87.5%) 8 (100%) 8 (100%) 6 (75%).
MAS-associated features: Intractable fever Hepatosplenomegaly Serositis Bleeding CNS involvement	8 (100%) 8 (100%) 5 (62.5%) 3 (37.5%) 2 (25%)
Hemoglobin, g\L (n.v. 120–145)	91 (83; 110)
White blood cells, x10^9^/L (n.v. 4.0–9.0 x 10^9^/L)	9.9 (2.1; 23.1)
Platelets, x10^9^/L (n.v. 150–350 x10^9^/L)	178 (127; 293)
C-reactive protein, mg/L (n.v. 0–5 mg/L)	73.8 (69.0; 81.8)
Ferritin, ng/mL (n.v. 15–120 ng/mL)	5.000 (3.157; 10.396)
Lactate dehydrogenase, U\L (125–220 U/L)	487 (323; 813)
Triglycerides, mmol/L (0.4–1.29 mmol/L)	2.85 (2.4; 3.0)
ALT, U\L (n.v. 0–55 U/L)	95 (36; 141)
AST, U\L (n.v. 0–34 U/L)	37 (28; 114)
GGT, U\L (n.v. 9–36 U/L)	73 (39; 163)
Initial biologic, *n* (%): Tocilizumab Canakinumab	3 (37.5) 5 (62.5)
Canakimumab, mg/kg (range)	4–25

*ALT, alanine aminotransferase; AST, aspartate aminotransferase; CI, confidence intervals; GGT, gamma-glutamyl transferase; MAS, macrophage activation syndrome*.

### Outcomes

Two sJIA patients (P1, P4) with MAS (Table 2), where tocilizumab was initiated, developed severe infusion reactions during the second dose and tocilizumab, prompting discontinuation. In a third patient (P8), tocilizumab was discontinued after 4 months due to disease flare (fever, rash) and inability to taper prednisolone to <10 mg daily. For all three patients, tocilizumab was switched to canakinumab.

**Table 2:** Treatments, prior to and following MAS resolution, and outcomes among sJIA patients who developed MAS.

**ID, Sex**	**JIA onset, years**	**Repeated MAS**	**Initial biologic**	**MAS on CAN**	**Experience of increased CAN doses**	**Outcomes**	**Current treatment**
1, F	7.7	N	TCZ	N	N	Remission	CAN
2, F	11.5	N	CAN	N	N	ILD	TCZ
3, F	7.9	Y	CAN	Y	Y	Remission	CAN
4, F	3.4	N	TCZ	N	Y	Remission, ILD	CAN, CS, MMF
5, M	14.2	Y	CAN	N	N	Remission	N
6, M	9.1	Y	psCS, IV CS, IVIG, IV CsA → CAN	N	N	Remission	N
7, M	1.3	N	CAN	Y	Y	Remission	CAN every 12 weeks
8, F	9.1	N	TCZ	Y	Y	Minimal disease activity	CAN, tofacitinib

*CAN, canakinumab; CS, corticosteroids; CsA, cyclosporine A; ID, identification; ILD, interstitial lung disease; IV, intravenous, IVIG, intravenous immunoglobuline; F,female; M, male; N, no; ps, pulse therapy; TCZ, tocilizumab; Y, yes*.

In patients where canakinumab was used as first-line therapy, the initial MAS episode in 3 patients (P2, P5, P6) completely resolved. In one child (P5) no subsequent episode of MAS was noted, but canakinumab was switched to tocilizumab due to incomplete sJIA control. This child is now in remission off all medication. P6 had a monocyclic sJIA disease course, initially treated with CS and methotrexate, achieving remission off all medications. However, P6 eventually developed severe MAS with profound pancytopenia, fever and remarkable hyper-ferritinemia (>20,000 ng/mL) and was transferred from his local rheumatological department to the ICU department of the center of pediatric hematology and transplantology. He had no response to high-dose CS (pulse therapy), IVIG and IV cyclosporine during 2 weeks, and was considered as a candidate for bone marrow transplantation He received a single canakinumab injection in addition to corticosteroids, cyclosporine, and antibiotics with gradual resolution of MAS during the following 4 weeks. Addition of canakinumab reversed the course of disease and allowed for successful tapering of CS and cyclosporine, but the patient's response was not as rapid compared to other children. Currently, P6 has been in remission off medication for more than 4 years.

P2 had severe sJIA with MAS and interstitial lung disease. She had minimal response to high-dose CS, IVIG, and cyclosporine, yet canakinumab administration achieved MAS remission. Her lung disease has been stable for 3 years, but canakinumab was switched to tocilizumab due to incomplete arthritis control despite absence of systemic features. Her arthritis remains under control on tocilizumab without CS.

Three patients developed sudden MAS during canakinumab treatment (P3, P7, P8). P7 developed MAS during the 3rd and 10th months of canakinumab and CS treatment. Both MAS episodes were severe (requiring ICU admissions) and were resistant to increased doses of CS (pulse therapy) and IVIG. Both severe MAS episodes required increased canakinumab dosing (150 mg per injection, 12 mg/kg) for MAS resolution. This child has trisomy 21 and had several milder MAS episodes prior to receiving canakinumab. During the last 8 years he has remained in remission without sJIA flares or MAS episodes while being treated with tapering doses of canakinumab (currently, 2 mg/kg every 12 weeks). A commercial primary immune deficiency panel DNA sequencing did not reveal any mutations in known familial hemophagocytic lymphohistiocytosis (HLH) or autoinflammatory disease - associated genes.

P3 was treated with canakinumab initially as part of a randomized clinical trial. Since the 2nd year of her diagnosis, she received 2 mg/kg of canakinumab every 4 weeks. During CS and canakinumab dose tapering, she developed MAS having been in remission for 2 years on a dosage of canakinumab of 2 mg/kg every 12 weeks. After failing increased CS and IVIG, the dose of canakinumab was increased (150 mg, ~5–6 mg/kg) to control the MAS. She has remained in remission of sJIA disease flares and MAS for 5 years on canakinumab dosed at 150 mg (3 mg/kg) every 4 weeks.

P8 developed several MAS episodes, required cyclosporine A and transient increasing of CS dose. Treatment with increased canakinumab dose (300 mg, ~ 7 mg/kg) did not prevent against MAS flare, but addition of the Janus kinase inhibitor tofacitinib (5 mg twice daily) to canakinumab (150 mg, 3 mg/kg monthly) achieved remission. Targeted DNA sequencing did not reveal any pathogenic/likely pathogenic variants in known familial HLH - or autoinflammatory disease - associated genes.

Four children with sJIA (P4, P7, P8) had multiple episodes of MAS. P4 first experienced severe (ICU setting) MAS at age 2 years with a poor response to CS and IVIG, and she experienced an infusion reaction to tocilizumab, prompting treatment with cyclosporine A and CS. After tapering CS over 1.5 years, she developed another severe sJIA flare and MAS episode without adequate response to high doses of CS, in addition to IVIG, cyclosporine A, and methotrexate. She received her first dose (Day1) of canakinumab (150 mg/kg, 7.5 mg/kg) with some improvement, but developed a flare within a week and again received 150 mg of canakinumab on Day 9 with complete resolution of all features of sJIA and MAS. The next canakinumab dose was on Day 28 (150 mg), followed by every 4 week dosing. The canakinumab treatment allowed for discontinuation of methotrexate and cyclosporine A. After 1.5 years of canakinumab treatment, she developed dyspnea, finger clubbing with distal phalanges erythema, and sJIA disease flare. Her chest CT and echocardiography revealed interstitial lung disease with mild pulmonary artery hypertension (25 mm Hg). She continued to receive 150 mg canakinumab (6 mg/kg) every 4 weeks, systemic CS dosing was increased, and cyclosporine A was added. A chest CT revealed disease progression, and a pulmonary biopsy demonstrated pulmonary alveolar proteinosis. Her diffusing lung capacity for carbon monoxide (DLCO) gradually decreased to 41%, and cyclosporine A was switched to mycophenolate mofetil. Azythromycin prophylaxis was also added. Over the next year, her DLCO improved to 70% and her pulmonary artery pressure normalized allowing for CS tapering to 4 mg of methylprednisolone daily, while continuing canakinumab and mycophenolate mofetil. Targeted sequencing of PID genes revealed a heterozygous *UNC13D* c.811C>T (p.Pro271Ser) variant (rs139564938, MAF = 0.000507); the variant was inherited from an asymptomatic father. Heterozygous mutations have been associated with familial HLH genes, including *UNC13D*, in children with systemic JIA and may contribute to MAS pathology (14, 15).

## Discussion

sJIA is an autoinflammatory and autoimmune condition with a peak age of onset of 2 years of age (16) but can occur in the first 12 months of life like P5 reported herein. MAS is a life-threatening feature of sJIA (2) and the major mortality factor, and MAS is likely more common in children with sJIA than previously considered (1, 17). Diagnostically, MAS can be a challenge as many features are shared with sJIA disease flares, and MAS can be present at disease onset (14). Unlike the HLH criteria (3), current MAS criteria for sJIA include liver dysfunction (15), as was noted in all children reported herein. Typically, elevated serum ferritin is a sensitive marker of MAS, as well new biomarkers such as IL-18, CXCL9, interferon-γ, and adenosine deaminase 2 (18). However, with the increased use of IL-1 and IL-6 blockade in children with sJIA, the ferritin value may be blunted, even in the setting of MAS, and assessment of the mentioned above biomarkers may be of greater diagnostic utility (19).

For many sJIA patients, IL-1 is a one of the main cytokines in the pathogenesis of sJIA (20) and associated MAS (2), and disease flares have been associated with increased amounts of pro-inflammatory cytokines, particularly IL-1β. Theoretically, increased IL-1 production can overwhelm the body's countering mechanisms (e.g., production of IL-1Ra). Anakinra is a recombinant version of naturally occurring IL-1Ra, and increased doses of IL-1Ra may be needed to counter excessive amounts of IL-1β. There are now several publications demonstrating the efficacy of increased anakinra dosing to treat MAS episodes (18). Despite its documented benefit in treating sJIA (21), anakinra is not available in all parts of the world. However, two other IL-1 blocking agents have been studied as therapy for sJIA, canakinumab (a monoclonal antibody to IL-1β) (8) and rilonacept (an IL-1 receptor fusion protein) (22).

Currently, there are limited data regarding the use of canakinumab and rilonacept for treating MAS in the setting of sJIA (23–25). Indeed, there are only a few cases of MAS reported to occur during or after canakinumab treatment of sJIA in randomized controlled trials (8). Herein, we report the use of increased doses of canakinumab in children with sJIA who initially were treated with standard doses of canakinumab for treatment of MAS. Several sJIA patients received one scheduled injection of an increased dose of canakinumab (150 mg, ~3- and 2-times standard doses, respectively) with rapid resolution of MAS allowing for tapering back down to standard canakinumab dosing of 4 mg/kg every 4 weeks. There were no notable short-term adverse events consistent with the published safety and efficacy of high doses of canakinumab from clinical trials. For example, after a single dose of 10 mg/kg of canakinumab, free IL-1β was reduced by more than 90% for more than 60 days (26). In a subsequent long-term phase III extension trial, children with cryopyrin gene mutations who experienced residual symptoms after the first canakinumab dose, responded to an increased dose of up to 8 mg/kg and/or shorter dosing frequency (27). As cytokine inhibitors target circulating or locally produced cytokines, there appears to be a larger therapeutic window for dosing of these medications compared to traditional medicine, which often directly target cells. One must also be cautious to not always attribute (cause and effect) the development of MAS in the setting of cytokine inhibition to the therapy itself, as increased dosing of the same biologic medication may be highly beneficial in resolving the MAS. Moreover, one of the patients resolved sJIA associated lung disease and pulmonary hypertension while remaining on canakinumab despite reports linking biologic therapies to the development of these sJIA features (28).

Although increased canakinumab dosing resolved the MAS in all 9 sJIA patients, there are some limitations to this report. Primarily, this was a retrospective cohort, which suffers from multiple potential biases and confounders not present in randomized, double blind, controlled trials. There were also multiple therapeutics used to treat these children complicating the interpretation of the outcomes. Nonetheless, while not proving direct cause and effect, for each child the increased dosing of canakinumab was followed by MAS resolution. Future prospective clinical trials are needed to demonstrate the benefit of increased canakinumab dosing to resolve MAS in the setting of sJIA.

## Conclusion

sJIA and MAS are often both IL-1-driven conditions. MAS can occur in the setting of sJIA treated with standard dosing of IL-1 blockade by canakinumab. This report provides evidence for the efficacy and safety of short-term increased doses (2–3-times normal) of canakinumab in treating sJIA associated MAS. Further study of the efficacy and safety of increased doses of canakinumab for treatment of MAS in children with sJIA is warranted.

## Data availability statement

The original contributions presented in the study are included in the article/supplementary material, further inquiries can be directed to the corresponding author/s.

## Ethics statement

The studies involving human participants were reviewed and approved by the Ethics Committee of Saint-Petersburg State Pediatric Medical University. Written informed consent to participate in this study was provided by the participants' legal guardian/next of kin.

## Author contributions

All authors listed have made a substantial, direct, and intellectual contribution to the work and approved it for publication.

## Funding

The work was supported by Russian Science Foundation grant 20-45-01005.

## Conflict of interest

Author RC has served as a consultant to SOBI, Novartis, Pfizer, and Sironax, and has received grant support from SOBI. The remaining authors declare that the research was conducted in the absence of any commercial or financial relationships that could be construed as a potential conflict of interest.

## Publisher's note

All claims expressed in this article are solely those of the authors and do not necessarily represent those of their affiliated organizations, or those of the publisher, the editors and the reviewers. Any product that may be evaluated in this article, or claim that may be made by its manufacturer, is not guaranteed or endorsed by the publisher.

## Abbreviations

ALT: alanine aminotransferase
AST: aspartate aminotransferase
CAN: canakinumab
CRP: C-reactive protein
CS: corticosteroids
CsA: cyclosporine A
ESR: erythrocyte sedimentation rate
FHLH: familial hemophagocytic lymphohistiocytosis
GGT: γ-glutamyl transferase
HLH: hemophagocytic lymphohistiocytosis
ID: identification
IL-1: interleukin-1
ILD: interstitial lung disease
IVIG: intravenous immunoglobulin
F: female
M: male
MAF: minor allele frequency
MAS: macrophage activation syndrome
MMF: mycophenolate mofetil
N: no
PID: primary immunodeficiency
sJIA: systemic juvenile idiopathic arthritis
TCZ: tocilizumab
Y: yes.
